# Mature Peripheral RPE Cells Have an Intrinsic Capacity to Proliferate; A Potential Regulatory Mechanism for Age-Related Cell Loss

**DOI:** 10.1371/journal.pone.0018921

**Published:** 2011-04-22

**Authors:** Ioannis Kokkinopoulos, Golnaz Shahabi, Alan Colman, Glen Jeffery

**Affiliations:** 1 Institute of Ophthalmology, University College London, London, United Kingdom; 2 School of Biomedical and Health Sciences Wolfson Centre for Age-Related Diseases, King's College London, London, United Kingdom; 3 Singapore Stem Cell Consortium, Singapore, Singapore; Center for Regenerative Therapies Dresden, Germany

## Abstract

**Background:**

Mammalian peripheral retinal pigmented epithelium (RPE) cells proliferate throughout life, while central cells are senescent. It is thought that some peripheral cells migrate centrally to correct age-related central RPE loss.

**Methodology/Principal Findings:**

We ask whether this proliferative capacity is intrinsic to such cells and whether cells located centrally produce diffusible signals imposing senescence upon the former once migrated. We also ask whether there are regional differences in expression patterns of key genes involved in these features between the centre and the periphery in vivo and in vitro. Low density RPE cultures obtained from adult mice revealed significantly greater levels of proliferation when derived from peripheral compared to central tissue, but this significance declined with increasing culture density. Further, exposure to centrally conditioned media had no influence on proliferation in peripheral RPE cell cultures at the concentrations examined. Central cells expressed significantly higher levels of E-Cadherin revealing a tighter cell adhesion than in the peripheral regions. Fluorescence-labelled staining for E-Cadherin, F-actin and ZO-1 in vivo revealed different patterns with significantly increased expression on central RPE cells than those in the periphery or differences in junctional morphology. A range of other genes were investigated both in vivo and in vitro associated with RPE proliferation in order to identify gene expression differences between the centre and the periphery. Specifically, the cell cycle inhibitor p27^Kip1^ was significantly elevated in central senescent regions in vivo and mTOR, associated with RPE cell senescence, was significantly elevated in the centre in comparison to the periphery.

**Conclusions:**

These data show that the proliferative capacity of peripheral RPE cells is intrinsic and cell-autonomous in adult mice. These differences between centre and periphery are reflected in distinct patterns in junctional markers. The regional proliferation differences may be inversely dependent to cell-cell contact.

## Introduction

The retinal pigmented epithelium (RPE) is a monolayer wrapping around the outer retina and forms part of the blood retinal barrier. It is critical for normal development of the adjacent neural retina [1] and at maturity sustains outer retinal function [2], [3], [4]. Both the RPE and the neural retina develop with a centre to periphery gradient, such that the last cells to leave the cell cycle are in the periphery [5], [6]. The far periphery is also thought to be a region from which stem cells can be harvested in the adult [7]. Mature peripheral RPE cells retain an ability to divide throughout life and some migrate into central senescent regions [8], [9]. Here we ask whether these peripheral cells have an intrinsic capacity to divide, marking them as distinct, or whether this ability is related to their local microenvironment and/or its signals. We examine RPE proliferative abilities in vitro from cells harvested from central and peripheral retinal regions and determine if central cells are able to impose senescence upon peripheral cells via soluble signals by exposing them to medium conditioned centrally. We also ask whether there is differential expression between the centre and periphery of key targeted genes involved in cell cycle activity and cell migration that may underpin their different abilities. In some non-mammalian vertebrates, RPE proliferation can be a step towards trans-differentiation and the building of a new retina, although such abilities have not been explored in mammals [10]. The long term aim of this study is to identify the key factors that distinguish those RPE cells that are able to trans-differentiate from those that are not.

## Results

### Peripheral RPE cells proliferate in vivo

**Figure 1:**
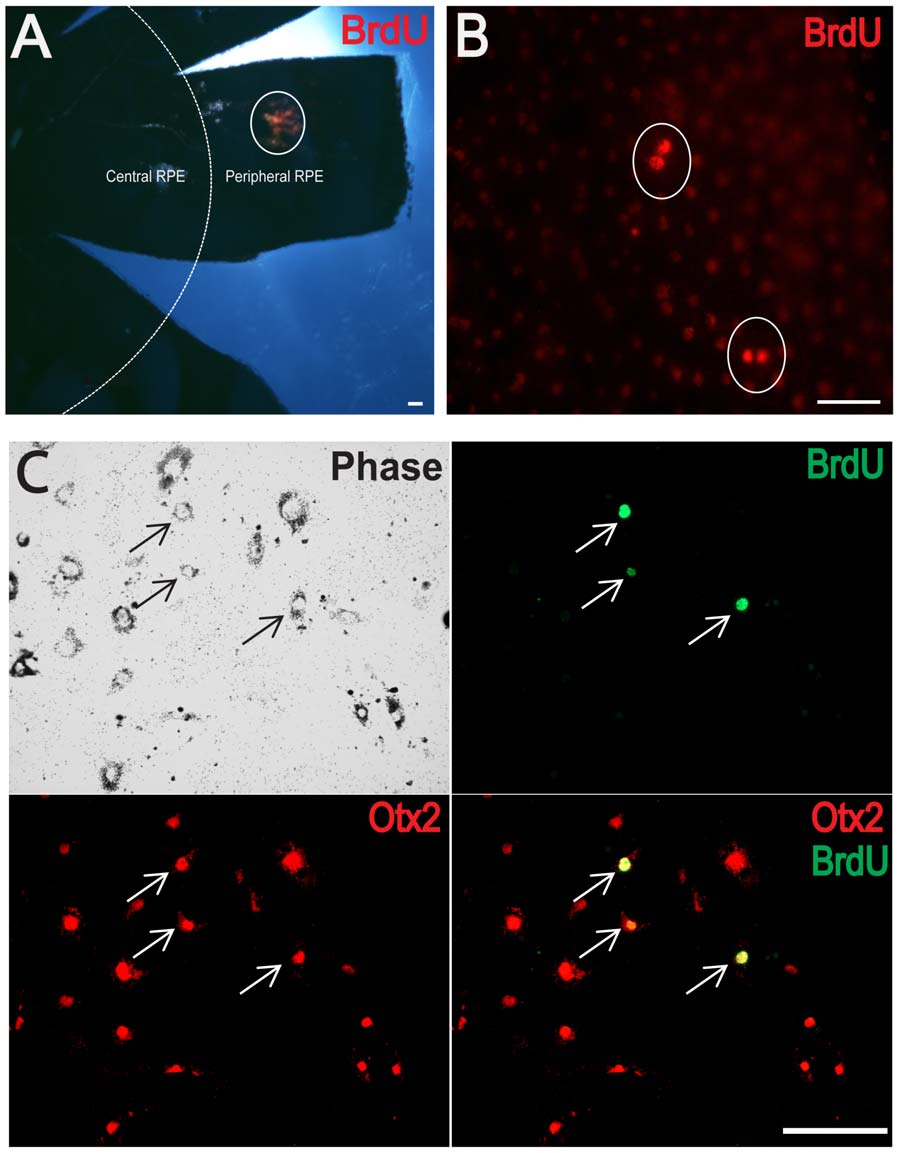
Peripheral RPE cells proliferate in vivo and in vitro. The adult mouse RPE was investigated to confirm it contains a peripheral region where cell division occurs. Animals were given daily BrdU injections for 5 consecutive days and were sacrificed 1, 2, 3 and 4 weeks after the last injection day. (A) Photomicrograph of the RPE flatmount indicating, in the circle, the peripheral region in which BrdU^+^ RPE cells were found. The dotted white line illustrates the peripheral and central RPE zones. (B) Higher magnification showing cells positive for BrdU (in red). (C) RPE cells were cultured for 9 days and on final day they received a single 4-hour BrdU pulse prior to fixation. The majority of cells expressed Otx2 (RPE-specific marker, red), with a number of cells also co-expressing BrdU (green). Scale bar = 5 µm. N (number of eyes examined) = 3.

Mouse RPE was examined following in vivo BrdU injections to confirm that RPE cells proliferate in this species. BrdU positive cells were present, but only in the periphery, consistent with previous studies [9] (Figure 1A, 1B). The only difference between the current results and previous studies was that fewer cells were identified in mouse than rat.

### Peripheral RPE cells proliferate more than central RPE cells in vitro

**Figure 2:**
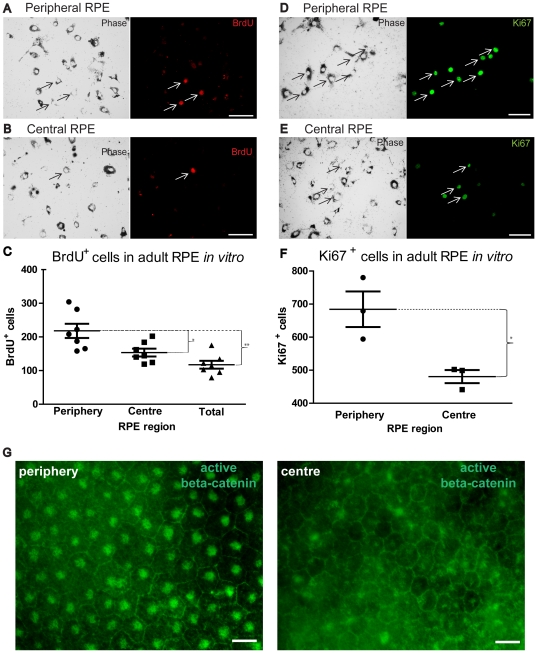
Peripheral, central and total RPE cell cultures have different proliferation profiles. Peripheral, central and total RPE cells were cultured for 9 days then received a single 4-hour BrdU pulse prior to fixation. Representative microphotographs of BrdU-labelled cultures of (A) peripheral RPE cells and (B) central RPE cells. White arrows indicate pigmented BrdU^+^ cells. (C) A graph indicating the number of BrdU^+^ cells in cultures harvested from each region (Peripheral: circles. Central: squares. Total: triangles). Mann-Whitney Test; *p = 0.02, **p = 0.04. No statistical significance was found between central and total RPE BrdU^+^ cells counts. Error bars = SEM. Peripheral and central RPE cells were cultured as above for 3 days, fixed and immuno-stained with Ki67. Representative microphotographs of Ki67^+^ cells of (D) peripheral cells and (E) central cells. White arrows indicate Ki67^+^ cells. The number of Ki67^+^ cells counted from each region is shown in Figure 2F where cell densities in the two cultures were similar (Periphery: circle points. Central; square points). Mann-Whitney Test; *p = 0.05. Error bars = SD. In A–C, when labelled cell numbers are normalised against the total cell number for the two regions the differences remain significantly different (Mann-Whitney Test; BrdU^+^/total cell number - Periphery against Centre; p = 0.037 and Ki67^+^/total cell number - Periphery against Centre; p = 0.0237). In vivo protein expression of active (phosphorylated) beta-catenin in peripheral and central RPE (G). N = 3 eyes. Scale bars = 5 µm.

The vast majority of cultured cells, irrespective of origin, were positive for Otx2 [11], confirming their identity as RPE cells (Figure 1C), with some co-expressing BrdU. All BrdU^+^ cells were also Otx2^+^. Low density RPE cultures were established from central and peripheral retinal regions and from the complete RPE sheet and exposed to BrdU. Significantly more proliferating cells were found in cultures derived from the periphery than the centre (Figure 2A, 2B, 2C). When whole RPE sheets were used and levels of proliferation assessed, they were different from those in the periphery but not from those from the centre (Figure 2C). Cells in these experiments had been cultured for 9 days and it is possible that differences in proliferation between central and peripheral areas were much smaller originally, but that over this period the pool of proliferating cells taken from peripheral regions expanded at a faster rate than those from the centre. When the number of BrdU cells is normalised against the total number for the two regions the differences remain significant. However, to confirm that differences between the two populations were genuine, cultures were run for only 3 days and then stained for the cell cycle marker Ki67 (Figure 2D, 2E). During this period the density of the two cultures remained the same. Again, differences in levels of proliferation between the two tissue groups remained significant with elevated cell cycle entry in cultures derived from peripheral regions (Figure 2F).

Beta catenin is a protein within adherent junctions in epithelial cells that is also part of the Wnt signalling pathway [12]. The latter pathway is implicated in epithelial cell growth and proliferation and the regulation of adherent junctions [13]. Membrane staining of beta catenin is associated with stable cell-cell adhesion, while the translocation of this protein to the nucleus is associated with cell proliferation [14]. Staining for the active form of beta-catenin in whole eye cups revealed different patterns between central and peripheral regions. In the central retina staining was relatively light, but mainly present in the cell membrane, while in the periphery staining was primarily nuclear (Figure 2G). Taken together the data presented in Figure 2 are consistent with the notion that there marked differences in the proliferative ability of the RPE between central and peripheral regions of the mature retina.

### RPE cell proliferation capacity is intrinsic and cell-contact dependent

**Figure 3:**
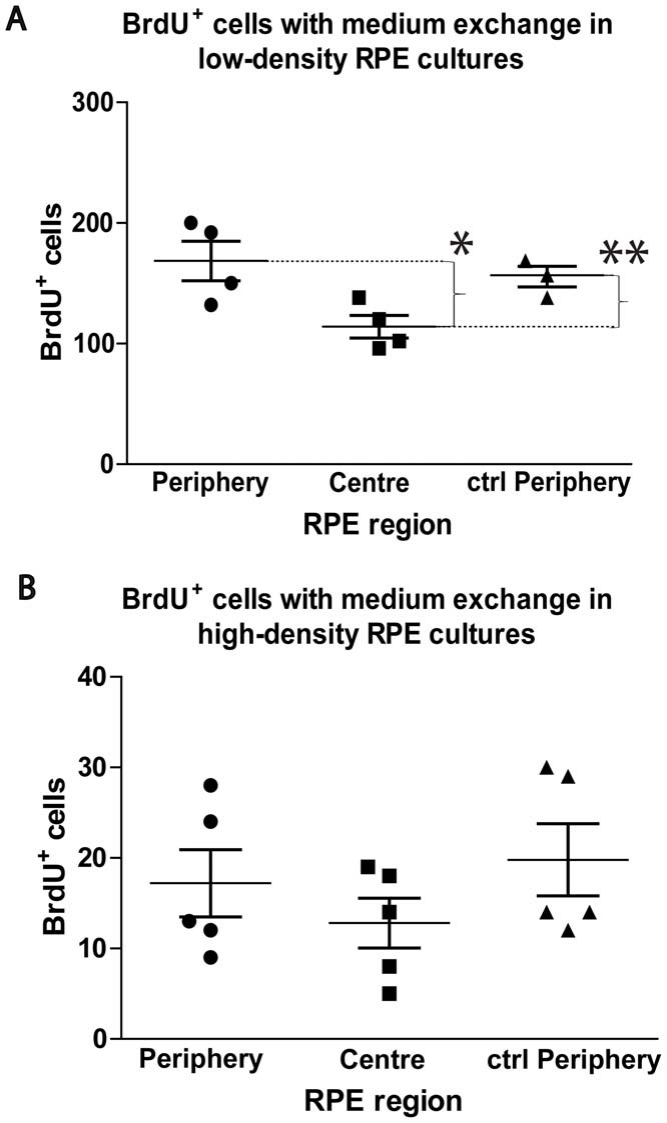
Central RPE cells do not inhibit peripheral RPE cells from proliferating via diffusible signals. RPE cultures were setup from peripheral and central RPE and were allowed to proliferate at low density for one week, before peripheral RPE cultures were introduced to central RPE medium for two days before a 4-hour BrdU pulse. (A) The graph indicates the number of BrdU^+^ cells per region per total cell number. Periphery (circle points), central (square points) and control periphery (triangle points). Cell number per culture; 5,000 cells/well. Mann-Whitney Test; *p = 0.03. **p = 0.02. Peripheral RPE versus control RPE periphery was not found to be statistically significant (p = 0.68). Error bars = SD. (B) RPE cultures were setup from peripheral and central RPE and allowed to proliferate at high density for one week, before peripheral RPE cultures were introduced to central RPE medium for two days before a 4-hour BrdU pulse. The graph indicates the number of BrdU^+^ cells per region. Periphery (circle points), central (square points) and control periphery (triangle points). Cell number per culture; 10,000 cells/well. Error bars = SD.

To determine if central cells express soluble agents suppressing proliferation, medium from their cultures was transferred to cultures of peripheral RPE cells (Figure 3). This was undertaken at the low density (Figure 3A) used above and at a higher (double) density (Figure 3B). Exchanging the medium at the normal density resulted in no difference in the proliferation rates of peripheral cells. When cell density doubled there was no difference in levels of proliferation between central and peripheral cells, but the increase in cell density reduced levels of proliferation by a factor of approximately 10 in all cell populations.

### Peripheral and central RPE cells express different gene patterns

**Figure 4:**
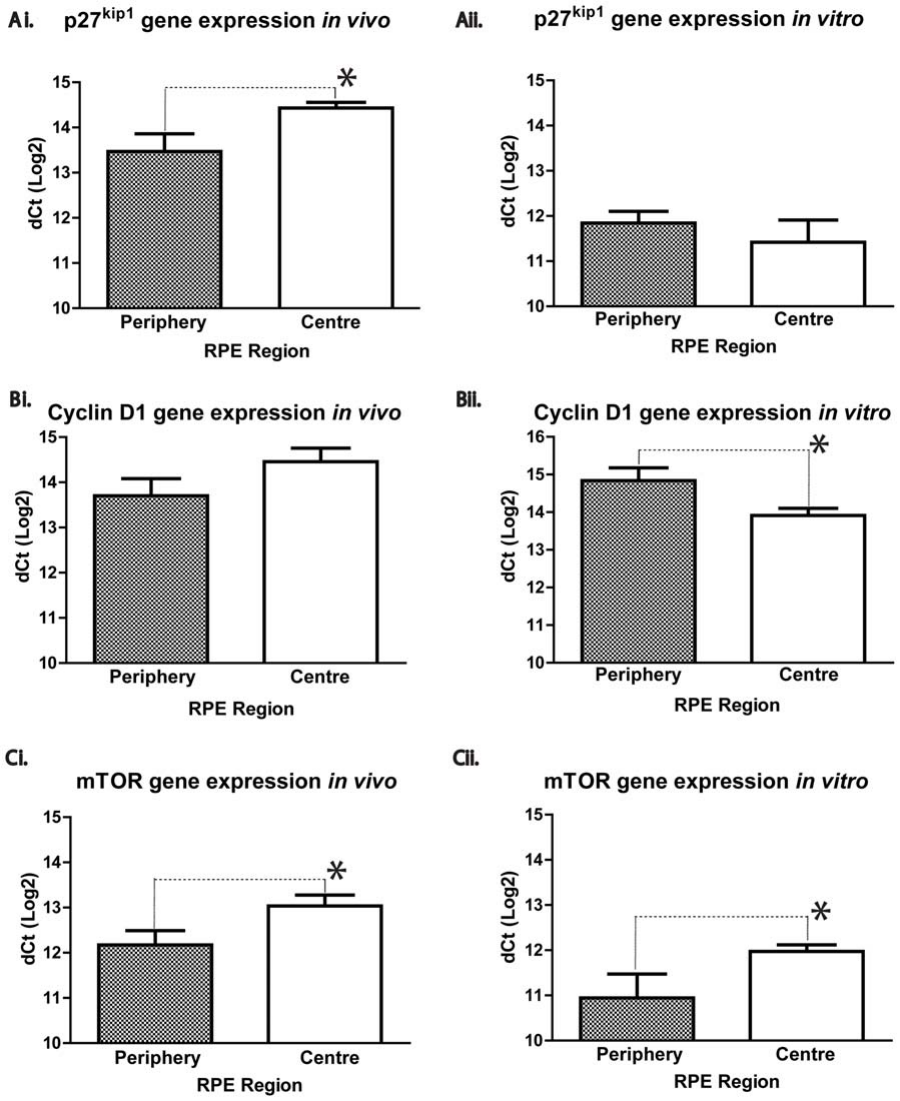
Quantitative Real-Time PCR analysis of gene expression in peripheral and central RPE tissue and cell cultures. Gene expression of p27^kip1^ (Ai.), Cyclin D1 (Bi.), and mTOR (Ci.), in RPE tissue obtained from the periphery and centre of adult mouse RPE. Gene expression of p27^kip1^ (Aii.), Cyclin D1 (Bii.), and mTOR (Cii.) in cultured cells obtained from the periphery and centre of adult mouse RPE. Graphs show relative gene expression levels from independent samples normalized to ACTB (at least 6 independent samples obtained from each region); Mann-Whitney Test, * P<0.05 in each case.

Gene expression analysis was used to investigate differences between peripheral and central RPE cells in vivo after culturing them for 9 days. The gene expression assays used were p27^Kip1^ (Figure 4A, n = 7–9, 5A, n = 5–6), Cyclin D1 (Figure 4B, n = 6–10, 5D, n = 5) and mTOR (Figure 4C, n = 4–8, 5B, n = 5) both in vitro and in vivo.

The cell cycle inhibitor p27^Kip1^ is expressed in the RPE [15], [16]. Quantitative Real-time PCR demonstrated significant differences in the expression of this gene between centre and peripheral regions, with significantly elevated gene expression levels in central RPE in vivo (Figure 4Ai p = 0.04). However, this difference was lost when the same analysis was undertaken on cells from these regions that had been maintained in low density cultures for 9 days (Figure 4Aii). Cyclin D1 is a cell cycle activator present in the RPE [17]. When the same analysis was undertaken on this gene, significant differences were only present in vitro with reductions found centrally (Figures 4Bi, 4Bii, p = 0.04). mTOR is involved in cell senescence [18]. Blocking mTOR in RPE cells reduces cell senescence leading to cell quiescence [19]. Lower mTOR expression levels may be associated with a cell's ability to initiate cell division. Real-time PCR data for mTOR demonstrated significant reductions in the level of its expression in peripheral cells in comparison to central RPE cells both in vivo and in vitro (Figures 4Ci, p = 0.03 & 4Cii, p = 0.04). Tgf-beta 3, which plays a role in cell senescence [20] was expressed on similar levels between central and peripheral RPE cultures and in vivo and in vitro (data not shown).

### Peripheral and central RPE cells have different junctional characteristics

**Figure 5:**
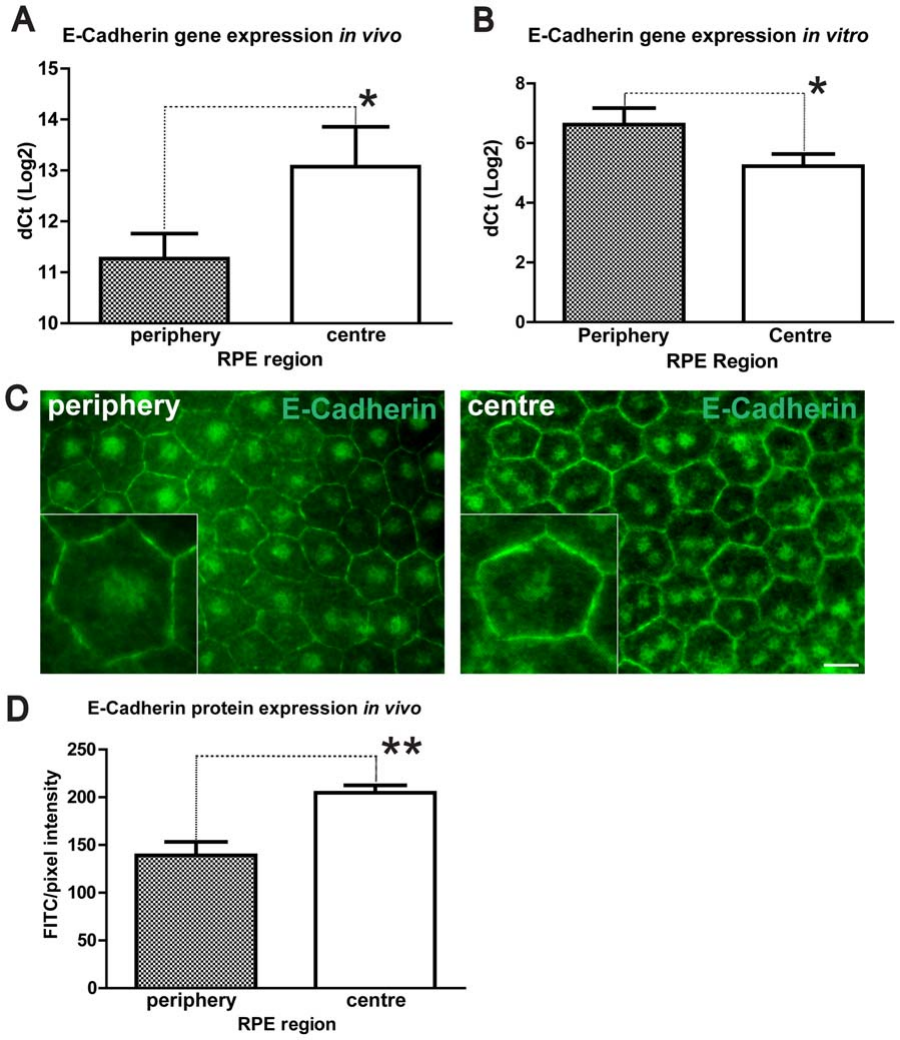
Gene and protein expression profile in peripheral and central RPE tissue of E-Cadherin. Gene expression of E-Cadherin, in RPE tissue obtained from the periphery and centre of adult mouse RPE in vivo (A) and in vitro (B). Graph shows relative expression levels from independent samples normalized to ACTB (n is at least 4 from each region). Protein expression of E-Cadherin (FITC) on adult mouse RPE flatmounts (C) and a graph showing FITC/pixel intensity from each RPE region (D). Central cells consistently expressed greater levels of E-Cadherin than those in the periphery. Inserts show individual RPE cells in higher magnification. N = 3 eyes. Scale bar = 5 µm. Mann-Whitney Test, * P<0.05, **p<0.002. Error bars = SEM.

These results demonstrate that peripheral RPE cells have an intrinsic capacity to divide, and that may be independent of soluble signalling from the central retina. Further, that the capacity to divide may be regulated by cell density. Given these differences, it is possible that there may also be different membrane protein characteristic between central and peripheral retinal regions. This is because cell addition within the established matrix will require established cells to shift to accommodate this change. E-Cadherin is a transmembrane protein that plays a key role in cell adhesion. Consequently, the levels of gene and protein expression between centre and periphery may differ, as peripheral cells may be more loosely packed or able to adjust their junctions, due to cell production in this area. Real-time PCR from in vivo tissue confirmed that significantly greater levels of the gene were present in vivo (Figure 5A, 5C–D). This pattern was reversed in vitro, perhaps because peripheral RPE proliferated at a higher rate and resulted in elevated cell density (Figure 5B). Differences in E-Cadherin patterns were also confirmed in vivo. Staining patterns for this protein were much brighter in central regions than in the periphery. This was obvious, and was confirmed quantitatively (Figure 5C, 5D). Hence, this junctional protein is expressed at lower levels in regions where cell addition takes place.

**Figure 6:**
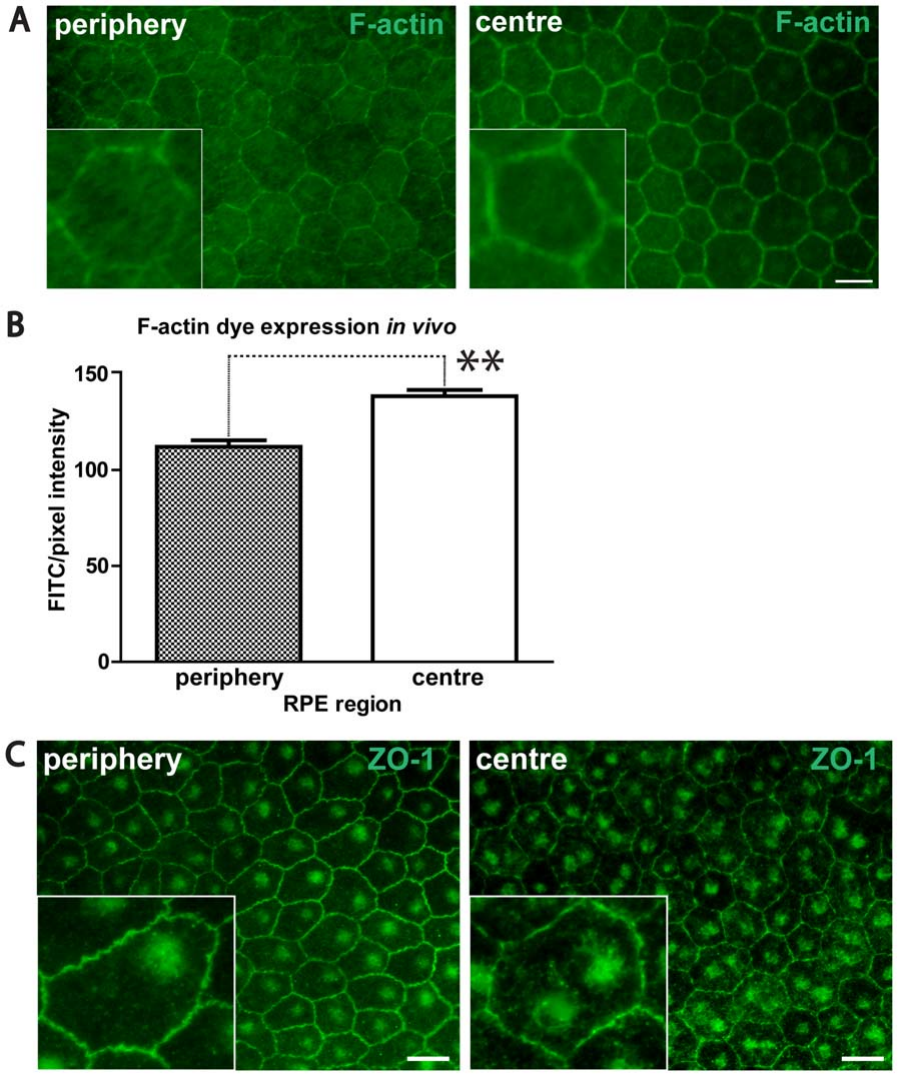
Protein expression profile in peripheral and central RPE tissue of F-actin and ZO-1 junctional markers. Phalloidin (F-actin) dye (FITC) expression on adult mouse RPE flatmounts (A) and a graph showing FITC/pixel intensity from each RPE region (B). Greater levels of F-actin were present on junctions centrally than in the periphery. Protein expression profile of ZO-1 in peripheral and central RPE (C). Inserts show individual RPE cells in higher magnification. ZO-1 is distributed less regularly in the periphery that in the centre. N = 3 eyes. Scale bar = 5 µm. Mann-Whitney Test, **p<0.002. Error bars = SEM.

The expression of other tight junction markers were also assessed between peripheral and central regions (Figure 6). Phalloidin marks F-Actin, which is associated with cell junctions [21]. Again, there were significant differences in the intensity of this label, with it being less well marked in the periphery than in the centre (Figure 6A, 6B). Further, Zonula Occludens Protein 1 (ZO-1) is expressed on RPE cells and is involved in cell density and epithelial cell proliferation control [22], [23], [24]. Staining for ZO-1 showed marked differences between central and peripheral regions. However, here the key difference was not the intensity of the label, but how regular it was distributed along the membrane surface. While this was relatively straight in central regions, in the periphery the label was consistently ruffled and irregular (Figure 6C).

## Discussion

This study shows differences in the rates of proliferation between central and peripheral RPE with elevated levels in peripheral regions that are likely to be cell autonomous. Culture media exchange from central to peripheral tissues failed to suppress this difference, providing evidence that at the concentrations applied here, it is unlikely that soluble factors were capable of influencing this difference. Further, we demonstrated differences in the expression of specific genes that may be responsible for the different behaviour of these cells, and show that there are key differences in junctional protein concentration/organisation between central and peripheral regions that may be associated with differences in proliferation.

Proliferation in the mature rodent RPE was identified in peripheral regions and it has been proposed that some of these cells migrate centrally to compensate for age related cell loss [9], [25]. Although we have demonstrated that this proliferation was likely to be cell autonomous, some cells cultured from the central RPE did divide, indicating that mature RPE cells can re-enter the cell cycle in low density cultures. One possible explanation might be that dissociation to single cell level might initiate an injury response, which induces cell cycle entry. However, as it is likely that some proliferating RPE cells migrate centrally and there become senescent [9], it is also possible that some of these cells that have migrated from the periphery may be released from that state by being placed in low density cultures.

**Figure 7:**
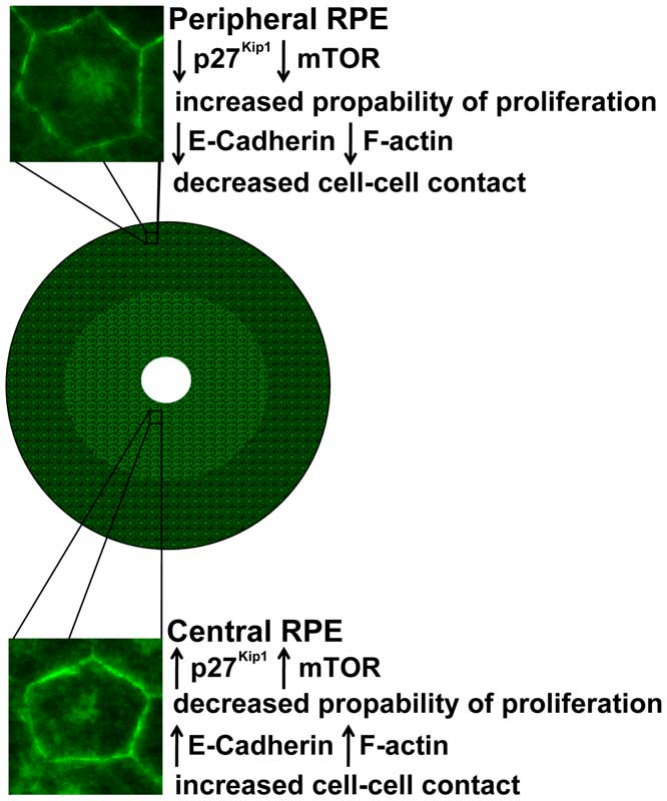
Schematic graph of the adult mouse RPE and characteristics of peripheral and central RPE cells. Peripheral and Central RPE cells express different levels of genes that play crucial role in cell proliferation. The levels of protein and gene expression for key cell adhesion molecules is also different between these two RPE populations, indicating distinct cell-cell contact behaviour.

Our gene expression analysis in vivo reveals that the adult mammalian RPE expresses genes encoding proteins that both augment and inhibit cell proliferation differentially between central and peripheral regions. mTOR [19], [26] regulates cell senescence [19] and was expressed less in the periphery, along with p27^kip1^, a cell cycle inhibitor [15], [16]. In contrast, Cyclin D1 and Tgf-beta 3 [27], [28] gene expression levels were similar between centre and periphery. When central and peripheral RPE cells were cultured separately some of the genes examined in vivo shifted their levels of expressions. Notably, there was a marked decrease in the gene expression of the cell cycle inhibitor, p27^Kip1^, in cultures, in comparison with their in vivo gene expression levels. Also, mTOR and Cyclin D1 gene expression levels between central and peripheral cultured cells in vivo and in vitro were different. However, overall gene expression profiles examined here generally supported the notion that at least two distinct RPE populations exist across the retina that may be correlated with differences in levels of cell autonomous proliferation. The differences identified here are represented diagrammatically in Figure 7.

Consistent with these regional differences in cell proliferation and gene expression, were the regional differences in junctional markers between central and peripheral retina. E-Cadherin, which is a key element in the RPE cell junction [29], [30], was lower in peripheral than in central RPE, which was reflected both in protein distribution and gene expression. Changes in RPE cell junctions were also confirmed by decreased F-actin staining on the junctional membrane in peripheral compared to central regions. While the levels of ZO-1 were similar in the centre compared to the periphery, junctions in peripheral regions were irregular, unlike those in the centre. Taken together, the most parsimonious explanation for the data presented here is that there are different genes regulating different proliferative abilities between central and peripheral regions, and that this in turn results in elevated peripheral cell division that loosens/modifies the junctions between cells.

If cells in a regular two-dimensional matrix proliferate the matrix in which they divide must change. There is strong evidence for this in the ageing human peripheral RPE. In young human retinae, the peripheral RPE appears as regular as that found in the centre. But by the early 20's the peripheral RPE has lost its regularity and many cells do not appear to be in a tightly ordered matrix [31]. If the peripheral RPE experiences life-long cell division at a relatively low level then this is exactly the pattern one would expect to find. That central RPE does not distort to the same extent with age implies that if there is cell migration, then this can only be happening to a limited proportion of the cells that divide.

Correlations between junctional modification and cell proliferation have been reported in a variety of epithelial tissues indicating, not surprisingly, that these events are associated. Studies have shown that changes in tight junctions are closely associated with cell proliferation and polarity via actin cytoskeletal modifications in intestinal epithelial cells [32], in mammalian tight junctions [33], in MDCK cells [24] and in human intestinal carcinomas [34]. In the latter, altered tight junction expression led to abnormal proliferation patterns. In these studies, elevated tight junction density is associated with decreased levels of proliferation. These results are consistent with the data presented here, where tight junctions appear to be modified in the retinal regions associated with proliferation.

The retina develops with a centre to periphery pattern, with the retinal margin being the last to mature [5], [6]. In amphibians and teleosts the retinal/RPE edge continues to produce new tissue throughout life [35] and the capacity of the peripheral mammalian RPE to proliferate may be a vestige of this. Excess RPE proliferation around the retinal margin occurs following retinal detachment [36], [37] and in response to local damage in a way that does not occur when similar events are imposed centrally [38]. In some amphibians retinal detachment not only results in RPE proliferations but also trans-differentiation into neural tissue [39], [40].

Taken together, the results presented here show that a population of peripheral RPE cells have a distinct ability to divide in a cell autonomous manner. We propose that the most likely signal(s) that inhibit further division in the central RPE probably arises from cell-cell contact. It would be interesting to assess whether direct contact between segregated central with peripheral RPE cells could alter the proliferation or migratory abilities of the latter.

## Materials and Methods

### Animals – Ethics Statement

Mice were maintained at University College London. All experiments were conducted in accordance with local and national British Home Office regulations Animals Scientific Procedures Act (1986). C57BL/6 mice were used throughout (2–3 months old). All animals were used with University College London ethics committee approval and under a UK Home Office project licence (PPL 70/6571).

### RPE cultures

Adult animals were killed by exposure to CO_2_. Eyes were placed in oxygenated L-15 medium (Invitrogen, UK) and hemisected using forceps and 7 mm curved micro-scissors. The anterior of the eye was removed, along with the retina leaving the RPE attached to the posterior eye. Five to ten eyecups were placed into 1.5 ml of Dispase I (2 U/ml, Invitrogen, UK) and incubated at 37°C/5% CO_2_ for 45 minutes. The eyes were removed from the enzyme treatment and washed briefly in Phosphate-Buffered Saline (PBS, 1×), before digesting them in trypsin (2 mg/ml, Invitrogen, UK) for 45 minutes at 37°C/5% CO_2_. After double enzyme treatment, the RPE was divided into peripheral and central regions. The average diameter of a mature mouse RPE is 5.5 mm. The central RPE was defined as that around the optic nerve head up to a maximum diameter of 3.3 mm. The 2.2 mm distal to this was defined as peripheral. The RPE cells were washed off in a small volume of medium and the sheets of cells were triturated mechanically, using a pipette. These cells were pooled and harvested by gently centrifuging at 4,000 rpm for 5 minutes. Cell pellets were resuspended in 1 ml of cell culture medium and counted. Cells were resuspended in DMEM-F12 plus Glutamax™ (Invitrogen, UK) containing N2 supplement (1∶100, Invitrogen, UK), Penicillin-Streptomycin solution (1∶100, Invitrogen, UK) and 1% Fetal Bovine Serum (FBS, Invitrogen, UK). 5×10^3^ to 10^4^ cells were plated into each well of Poly-L-Lysine-coated 24-well tissue culture dishes (Sigma-Aldrich, UK) in a maximum of 350 µl of medium per well at low cell densities of 8 cells/mm^3^ and high cell densities of 15 cells/mm^3^. The medium was not changed on the cells for one week after harvesting. In the second week, half of the medium was exchanged with fresh one.

To determine if medium exchange influences cell proliferation, RPE cultures were setup from peripheral and central RPE and allowed to proliferate at low and high densities for one week, before peripheral RPE cultures were introduced to central RPE medium for two days before a 4-hour BrdU pulse. Central RPE cultures were replaced with fresh medium.

### Incorporation of bromodeoxyuridine (BrdU)

Incorporation of BrdU (Sigma-Aldrich, UK) in vitro during S-phase was used as an assay for cell proliferation. RPE cells from the adult mouse were cultured as described above. On day 7 in vitro, cells were pulse-labelled with BrdU (0.5 µM) and 1 µl (4 M) NaOH in DMEM-F12+Glutamax for 4 hours. Incorporation of BrdU in vivo during S-phase was used as described previously [8].

### Immunocytochemistry

Adherent cells were washed in PBS prior to fixation with 4% PFA for 10 minutes at Room Temperature (RT). Cells were washed 3×2 minutes in PBS (1×) and pre-blocked in 1× PBS containing FBS (1%), bovine serum albumin (BSA) (10%) and 0.5% Triton-X 100 for 2 hours at RT before being incubated with primary antibody in blocking solution overnight at RT. After rinsing 3×2 minutes with PBS, cells were incubated with secondary antibody for 1 hour at RT and rinsed (3×2 minutes) in 1× PBS. Cells were again fixed in 4% paraformaldehyde (PFA, Sigma-Aldrich, UK) for 10 minutes at RT and washed with 1× PBS (3×2 minutes), before treating with 6 M HCL for 30 minutes at RT. Cells were washed in 1× PBS (3×2 minutes), before blocking with 1× PBS containing FBS (1%), bovine serum albumin (BSA) (10%) and 0.5% Triton-X 100 for 2 hours at RT. Cells were briefly washed in 1× PBS and anti-BrdU was added using the same blocking solution and left overnight at RT. Cells were washed in 1× PBS (3×2 minutes), before the appropriate secondary antibody was added, diluted in the same blocking solution, for 2 hours at RT. Cells were washed in 1× PBS (3×2 minutes) and DAPI-Vectorshield (Vector Labs Ltd, UK) was added. Cells were stored at 4°C.

### Immunohistochemistry

Eyes were fixed in 4% PFA in PBS for 1 hour at RT and washed in 1× PBS. The cornea, lens and retina were removed from each eye. Incisions were made into the eye to make sure that the eye could be flatmounted. Flatmounts were washed 3 times in 1× PBS for 5 minutes each on a shaking machine, before blocking with 5% normal donkey serum (NDS) in 3% Triton X-100 in 1× PBS for 2 hours at RT. Flatmounts were briefly washed in 1× PBS, and incubated with the appropriate primary antibody overnight at RT. The next day, flatmounts were washed in 1× PBS before the appropriate fluorescent-conjugated secondary antibody was added in 1% NDS with 0.3% Triton X-100 for 2 hours at RT. The eyecups were washed in 1× PBS before being fixed again in 4% PFA for 10 minutes at RT. Antigen retrieval was carried out using 6 M HCl in 0.1% Triton X-100 for 30 minutes at RT. Flatmounts were washed in 1× PBS and then were blocked with 5% NDS in 3% Triton X-100 in 1× PBS for 2 hours at RT. Anti-BrdU was added in 1% NDS in 3% Triton X-100 in 1× PBS overnight at RT. After the tissue was washed in PBS, the appropriate secondary antibody was added in 1% NDS with 0.3% Triton X-100 for 2 hours at RT. Eyes were washed in 1× PBS and DAPI was added (1∶5,000) for 1 minute. Flatmounts were then washed in 1× PBS and 1× TBS, before being mounted with vectorshield and stored at 4°C.

### Measurement of FITC/pixel intensity

Fluorescence images of the area around TIFF format using a 40× objective lens and a 10× eyepiece, using an Epi-fluorescence bright-field microscope (Olympus BX50F4, Olympus, Japan) with an 24-bit colour images at 3840×3072 pixel resolution using Nikon DXM1200 (Nikon, Tokyo, Japan) digital camera. The pictures were put together and the integrated density which is the product of the area chosen (in pixels) and the mean gray value (the measurement of the brightness) were measured using Adobe Photoshop CS4 extended. The lasso tool was used to draw a line all the way around the RPE cells and the integrated density was measured.

### Antibodies

The following antibodies were used: anti-BrdU (mouse, 1∶5, gift from Marcus Fruttiger), Otx2 (rabbit; 1∶500; Millipore), E-Cadherin (mouse, 1∶500; BD Biosciences), Ki67 (rabbit, 1∶1000, Vector Labs), ZO-1 (rabbit, 1∶100, AbCam) and active Beta-Catenin (mouse, 1∶200, Upstate Labs). The appropriate Alexa-tagged secondary antibodies (Molecular Probes, Invitrogen) were used and nuclei were counterstained, where appropriate, with DAPI. Staining was visualized using a Leica Inverted (Leica DM IRB, Germany) fluorescence microscope.

### Real-Time Polymerase Chain Reaction (Re-Ti PCR)

Total RNA was extracted from RPE cells either directly obtained from RPE or after cultured for 9 days in vitro using the RNAeasy Isolation Kit (Invitrogen, UK) and converted to cDNA, according to the manufacturer's instructions. Samples were used as templates for quantitative real-time polymerase chain reaction (Re-Ti-PCR) on a 7500 Fast Real-Time PCR™ System, using TaqMan^R^ (Applied Biosystems, UK) gene expression assay kits for mouse p27^Kip1^ (Cdkn1b), Cyclin D1, mTOR, E-Cadherin and Tgf-beta 3, according to the manufacturer's protocol. Quantitative Real Time PCR (Re-Ti PCR) stages were: (i) denaturation stage, 1 cycle at 50°C for 2 m and 95°C for 10 m (ii) amplification stage, 40 cycles at 95°C for 15 s and 60°C for 1 m. Data collection was set at the amplification stage in each cycle. At least four independent samples of each region were analysed in duplicate TaqMan assays. Mouse ACTB gene expression was used as an endogenous control showing similar expression pattern between duplicates. Relative quantities of the target mRNAs were normalized against the endogenous control [by subtracting CT (threshold cycle) values for ACTB from CT values for all gene expression assays]. Relative expression values were calculated as 2^−ΔCT^ Fold (log2). Expression changes were calculated using the formula 20-2^−ΔCT^ as shown on Figures 4 and 5.

### Statistics

Results are presented as the mean ± SEM or ± SD. Where appropriate, ***n*** indicates the number of individual cell cultures or eyes investigated. Statistical analysis was performed using GraphPad Prism 5 using a Mann-Whitney's test.
